# Differential analysis of urinary stable downregulated proteins in male patients after colorectal cancer surgery

**DOI:** 10.3389/fonc.2026.1841166

**Published:** 2026-08-24

**Authors:** Lupeng Wang, Qian Meng, Mei Hu, Man Zhang

**Affiliations:** 1 Clinical Laboratory Medicine, Beijing Shijitan Hospital, Capital Medical University, Beijing, China; 2 Beijing Key Laboratory of Urinary Cellular Molecular Diagnostics, Beijing, China; 3 Institute of Regenerative Medicine and Laboratory Technology Innovation, Qingdao University, Qingdao, China

**Keywords:** biomarker, colorectal cancer, prognosis, protein expression, urine

## Abstract

**Background:**

Colorectal cancer is a common malignant tumor that threatens men’s health, and there are currently ineffective biomarkers for noninvasive monitoring of postoperative tumor changes in clinical practice. Urine is an easily accessible sample that can reveal physiological and pathological changes in the body. To identify specific proteins stably downregulated postoperatively and thereby provide novel potential biomarkers for colorectal cancer following surgery monitoring, this study analyzed urinary proteomic changes in male colorectal cancer patients before and after surgery.

**Methods:**

This study included 56 male colorectal cancer patients, separated into three groups according on urine sample collection timing: preoperative, postoperative 4 ± 1 day, and postoperative≥8 days. Label-free quantitative proteomics was used to examine urine protein expression profiles. Bioinformatics research revealed differently expressed proteins with a consistent decrease trend postoperatively. Their clinical diagnostic efficacy was assessed using correlation analysis and receiver operating characteristic (ROC) curves.

**Results:**

This study identified 10 proteins exhibiting a stable downward trend post-surgery through urinary proteomics analysis. Correlation analysis revealed a significant positive correlation between PSMA7 and IDH1 (*P* < 0.05), as well as between RPS3 and RACK1 (*P* < 0.05). The diagnostic performance of the four proteins (PSMA7, IDH1, RACK1, RPS3) was 0.888, 0.831, 0.823, and 0.805, respectively (all *P* < 0.05). The combined diagnostic model achieved an AUC of 0.912.

**Conclusion:**

The urinary levels of IDH1, PSMA7, RPS3, and RACK1 demonstrated stable downregulation across postoperative stages with certain diagnostic efficacy, and these proteins hold promise as candidate novel non-invasive biomarkers for auxiliary monitoring of urinary proteomic changes in male colorectal cancer patients following surgery. However, their clinical utility warrants further validation in large-scale independent cohorts.

## Introduction

Colorectal cancer (CRC) is the third most prevalent malignant tumor and the second major cause of cancer death globally (1–3). According to epidemiological data, there are notable gender differences in the incidence of colorectal cancer (CRC), with males having a significantly higher risk and a generally worse prognosis than females (4, 5). Therefore, in the preliminary exploratory phase of this study, male patients were preferentially enrolled to minimize the potential confounding effects of sex-related physiological differences on urinary proteomic profiles, thereby enhancing the consistency and comparability of inter-group analyses. Currently, extensive surgical resection is the cornerstone for long-term survival in CRC patients (6). However, surgical success is dependent not only on the removal of macroscopic lesions, but also on the successful clearance and monitoring of postoperative minimal residual disease (MRD) (7, 8). Early postoperative surveillance presents considerable problems for clinically used serum tumor indicators (such as CEA and CA19-9). Because of their wide half-life variability, limited specificity, and susceptibility to interference from non-specific inflammatory responses triggered by surgical trauma (9, 10), they struggle to accurately and specifically reflect the true dynamics of tumor burden during the critical recovery window post-surgery.

Urine is a suitable liquid biopsy sample (11), displaying particular benefits in this sector. Its gathering procedure is fully non-invasive, allowing for high-frequency continuous monitoring. Furthermore, urine composition is substantially less influenced by homeostatic mechanisms in the body, allowing for more direct enrichment and amplification of pathological changes. Urine is the final product created after blood is filtered, reabsorption, and secreted by the kidneys (12). It is rich in low-molecular-weight proteins and peptides derived from numerous tissue systems throughout the body, acting as a “molecular mirror” of the organism’s overall state. Urine proteomics has proven significant potential in discovering disease-associated biomarkers, as high-throughput mass spectrometry technology has rapidly advanced (13, 14).

The development and progression of tumors are accompanied by the remodeling of core metabolic characteristics. Colorectal cancer (CRC) cells maintain rapid proliferation through energy metabolism reprogramming (such as enhanced glycolysis) and alterations in protein synthesis or degradation (15, 16). Following surgical resection of the primary tumor, the tumor-driven abnormal metabolism and protein synthesis pathways are disrupted, allowing the body’s internal environment to gradually transition from a tumor-associated hypermetabolic state back toward physiological homeostasis (17, 18). This systemic recovery process is inevitably accompanied by a systematic decline in the levels of relevant metabolic enzymes and signaling proteins in blood and body fluids. These circulating tumor-associated proteins and their degradation fragments are processed by the kidneys and excreted into urine (19), making alterations in the urinary protein profile an ideal window for monitoring this dynamic recovery process.

Although previous studies have employed urinary proteomics to characterize differences between CRC patients and healthy individuals (20), systematic investigations focusing on the dynamic changes in urinary proteomic profiles across distinct stages before and after surgical resection remain lacking. In this study, a cohort of male patients with colorectal cancer was selected, and label-free quantitative proteomics was applied to comprehensively compare urinary protein expression profiles among three independent patient groups, representing the preoperative, early postoperative, and postoperative recovery stages, respectively. By characterizing the alterations in urinary proteomic profiles before and after CRC surgery, this study aimed to identify and validate proteins exhibiting a stable postoperative downregulation trend, and to preliminarily evaluate their potential clinical utility as noninvasive biomarkers for postoperative monitoring of colorectal cancer.

## Materials and methods

### Study subjects

This study recruited male colorectal cancer patients admitted to the Department of Gastrointestinal Surgery at Beijing Shijitan Hospital, Capital Medical University. Participants were divided into three independent cohorts based on the timing of urine sample collection relative to surgery: the preoperative colorectal cancer group (PRD, n=25), the postoperative 4 ± 1 day group (POD4, n=14), and the postoperative ≥8 day group (POD8, n=17). Exclusion criteria were: 1. Concurrent malignant tumors other than colorectal cancer; 2. Severe infectious diseases or systemic inflammatory response syndrome; 3. Prior antitumor therapy before surgery. All subjects provided written informed consent before study participation. This study protocol was reviewed and approved by the Ethics Review Committee of Beijing Shijitan Hospital.

### Sample collection and preparation

All subjects provided midstream urine samples (50–100 mL) collected in the morning on an empty stomach into sterile urine cups. Samples were immediately placed on ice and transported to the laboratory within 2 hours for pretreatment. Samples were centrifuged at 1,500 × g for 15 minutes at 4 °C to remove cellular debris, bacteria, and insoluble precipitates. After centrifugation, the supernatant was carefully aspirated, aliquoted into sterile storage tubes, and promptly transferred to a -80 °C freezer for storage. Care was taken throughout the procedure to strictly avoid repeated freeze-thaw cycles of the samples. Urine samples designated for proteomic analysis were subjected to total protein quantification using the bicinchoninic acid (BCA) assay, after which equal amounts of protein were taken for subsequent experiments. Protein samples were enzymatically digested with trypsin, and the resulting peptides were desalted using C18 solid-phase extraction, followed by vacuum drying in preparation for subsequent liquid chromatography-tandem mass spectrometry (LC-MS/MS) analysis.

### LC-MS/MS analysis

Load 1 μg of peptide sample onto a custom-made C18 analytical column (150 μm × 20 cm, packed with 1.9 μm ReproSil-Pur C18-AQ). Detection was performed using a Q-Exactive HF mass spectrometer (Thermo Fisher Scientific) in data-independent acquisition (DIA) mode. Liquid separation employed a 75-minute gradient elution at a flow rate of 600 nL/min. Primary mass spectrometry (MS1) resolution was set to 60,000 (scan range 300–2000 m/z). Secondary mass spectrometry (DIA MS/MS) resolution was 30,000, with 40 isolation windows covering the 400–1000 m/z range and normalized collision energy (NCE) set to 28. To minimize technical bias, all samples were processed using a standardized experimental workflow and identical instrumental parameters throughout the analysis.

### Protein identification and quantification

DIA raw data were processed using Spectronaut software (version 17.4; Biognosys) and searched against the UniProt human protein database. Search parameters were set as follows: Trypsin digestion with a maximum of 2 missed cleavage sites; fixed modifications included cystine N-formylation; variable modifications included methionine oxidation and N-terminal acetylation of proteins. The false discovery rate (FDR) was controlled below 1.0% at both the peptide and protein levels, and each protein required at least one unique peptide to be considered a valid identification. Relative quantification was performed using protein intensity values exported from Spectronaut. Differentially expressed proteins (DEPs) were defined by a fold change (FC) greater than 1.5 or less than 0.67, with a false discovery rate (FDR)-adjusted *P* value of less than 0.05.

### Statistical analysis

This study employed SPSS 27.0.1 and GraphPad Prism 9.5.0 for routine statistical analysis. Bioinformatics analysis was conducted using the R programming language, and relevant figures were generated with Origin 2024 software. Normally distributed quantitative data were expressed as mean ± standard deviation (mean ± SD), and comparisons among multiple groups were performed using one-way ANOVA. Non-normally distributed quantitative data were expressed as median (interquartile range) [M (IQR)], and intergroup comparisons were performed using the Mann-Whitney U test or Kruskal-Wallis H test. Correlations between variables were assessed using Spearman’s rank correlation analysis. To evaluate the diagnostic performance of candidate proteins for pre- and post-surgical states in colorectal cancer, receiver operating characteristic (ROC) curves were constructed, and the area under the curve (AUC), sensitivity, and specificity were calculated. A two-tailed *P* < 0.05 was considered statistically significant.

## Results

### Comparison of clinical data among the three groups

This study included a total of 56 cases for analysis: 25 cases in the PRD group, 14 cases in the POD4 group, and 17 cases in the POD8 group. Statistical comparisons were performed on baseline clinical data and laboratory test indicators among the three groups, as shown in **Table 1**. No statistically significant differences were observed among the three groups in age, white blood cell count (WBC), red blood cell count (RBC), hemoglobin (HGB), platelet count (PLT), liver function (ALT, AST, ALB), renal function (Urea, Cr, eGFR), or metabolic indicators (TC, TG, Glu) (*P*>0.05). Furthermore, no statistically significant differences were observed among the three groups with respect to clinicopathological characteristics including tumor location, pathological stage (pStage), and degree of differentiation (*P*>0.05). These results indicate that the three groups are comparable in baseline characteristics.

**Table 1:** Comparison of baseline clinical characteristics among three patient groups.

Indicators	PRD	POD4	POD8	*P* value
	N=25	N=14	N=17	
Age, years	65 ± 11	63 ± 12	63 ± 11	0.810
WBC, 10^9^/L	6.40 ± 2.00	6.48 ± 1.80	6.46 ± 2.14	0.993
RBC, 10^12^/L	4.38 ± 0.65	4.34 ± 0.77	4.23 ± 0.63	0.776
HGB, g/L	131 ± 23	129 ± 26	128 ± 21	0.905
PLT, 10^9^/L	240 ± 109	248 ± 131	250 ± 126	0.964
ALT, U/L	17 ± 6	17 ± 8	17 ± 7	0.925
AST, U/L	17 (15-20)	18 (13-20)	19 (15-21)	0.814
ALB, g/L	40.8 ± 4.8	40.7 ± 5.8	41.1 ± 4.9	0.972
Urea, mmol/L	5.21 (4.46-6.51)	4.85 (4.48-6.18)	5.28 (4.54-6.51)	0.796
Cr, µmol/L	76 ± 14	79 ± 11	77 ± 15	0.798
eGFR, mL/min/1.73 m^2^	90 ± 15	89 ± 13	90 ± 15	0.974
TC, mmol/L	4.36 ± 1.03	4.54 ± 1.07	4.20 ± 0.98	0.666
TG, mmol/L	1.30 (1.03-1.87)	1.37 (1.07-2.01)	1.36 (1.11-2.15)	0.889
Glu, mmol/L	5.87 ± 1.06	5.81 ± 1.20	5.82 ± 1.05	0.952
Location				0.889
Rectum	6 (24.0%)	5 (35.7%)	6 (35.3%)	
Colon	13 (52.0%)	7 (50.0%)	8 (47.1%)	
Rectosigmoid junction	6 (24.0%)	2 (14.3%)	3 (17.6%)	
pStage				0.993
Stage I	4 (16.0%)	2 (14.3%)	1 (5.9%)	
Stage II	10 (40.0%)	5 (35.7%)	10 (58.8%)	
Stage III	6 (24.0%)	4 (28.6%)	3 (17.6%)	
Stage IV	5 (20.0%)	3 (21.4%)	3 (17.6%)	
Differentiation				0.919
Well	2 (8.0%)	1 (7.1%)	1 (5.9%)	
Moderate	20 (80.0%)	12 (85.7%)	14 (82.4%)	
Moderate-poor	2 (8.0%)	0 (0.0%)	1 (5.9%)	
Poor	1 (4.0%)	1 (7.1%)	1 (5.9%)	

Data are presented as mean ± SD or median (IQR) or n (%). Non-normally distributed variables compared by Kruskal-Wallis test; all others compared by one-way ANOVA. Nominal categorical variable compared by Pearson chi-square test. Ordinal variables compared by Spearman rank correlation. *P* < 0.05 is considered statistically significant. WBC, white blood cell count; RBC, red blood cell count; HGB, hemoglobin; PLT, platelet count; ALT, alanine aminotransferase; AST, aspartate aminotransferase; ALB, albumin; Urea, blood urea nitrogen; Cr, creatinine; eGFR, estimated glomerular filtration rate; TC, total cholesterol; TG, triglycerides; Glu, blood glucose.

### Differential protein analysis between the PRD and POD4 group and the PRD and POD8 group

**Figure 1:**
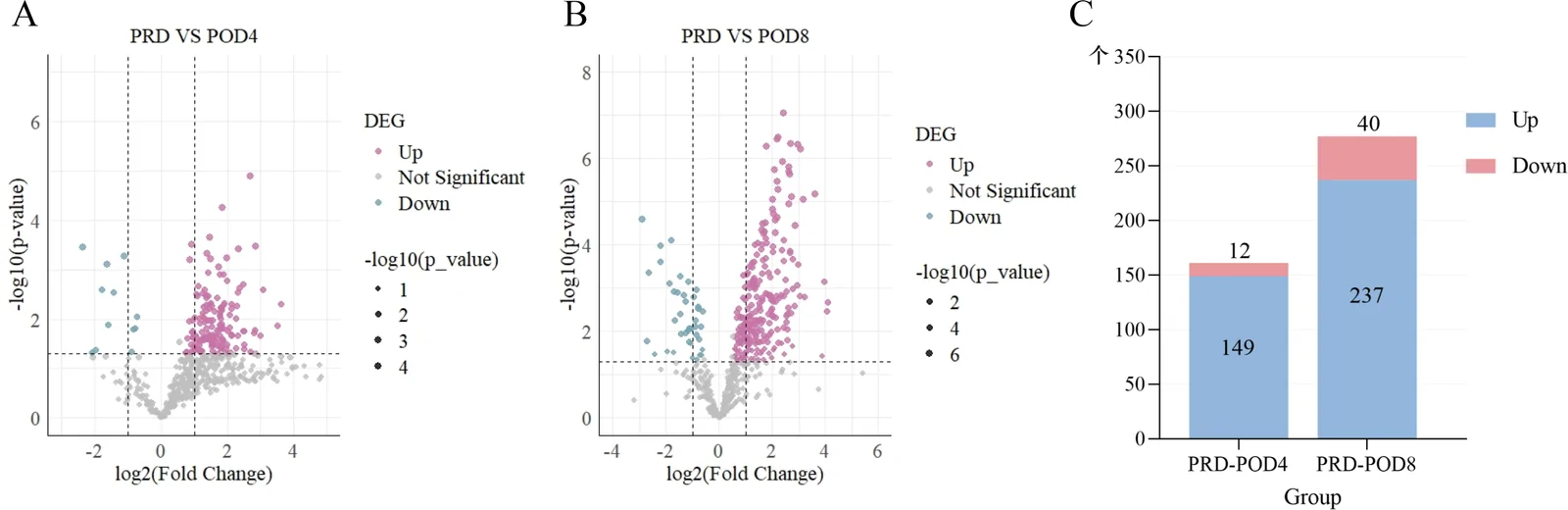
Analysis of differentially expressed proteins (DEPs) between different comparison groups. **(A)** PRD vs POD4 differential expression volcano plot. **(B)** PRD vs POD8 differential expression volcano plot. **(C)** Stacked bar chart of DEPs distribution across different comparison groups.

Label-free quantitative proteomics analysis of urine samples collected from three groups of colorectal cancer patients—preoperative (PRD), early postoperative (POD4), and recovery phase (POD8)—identified a total of 3,695 proteins, with 544 proteins common across all three groups. Significance was defined as fold change (FC) >1.5 or <0.67 with *P* < 0.05. Volcano plot analysis (**Figures 1A, B**) revealed 161 differential expression proteins (DEPs) between the PRD and POD4 groups, comprising 149 upregulated and 12 downregulated proteins. 277 DEPs were identified between the PRD and POD8 groups, comprising 237 upregulated and 40 downregulated proteins (**Figure 1C**).

### Mfuzz clustering analysis of differentially expressed proteins

**Figure 2:**
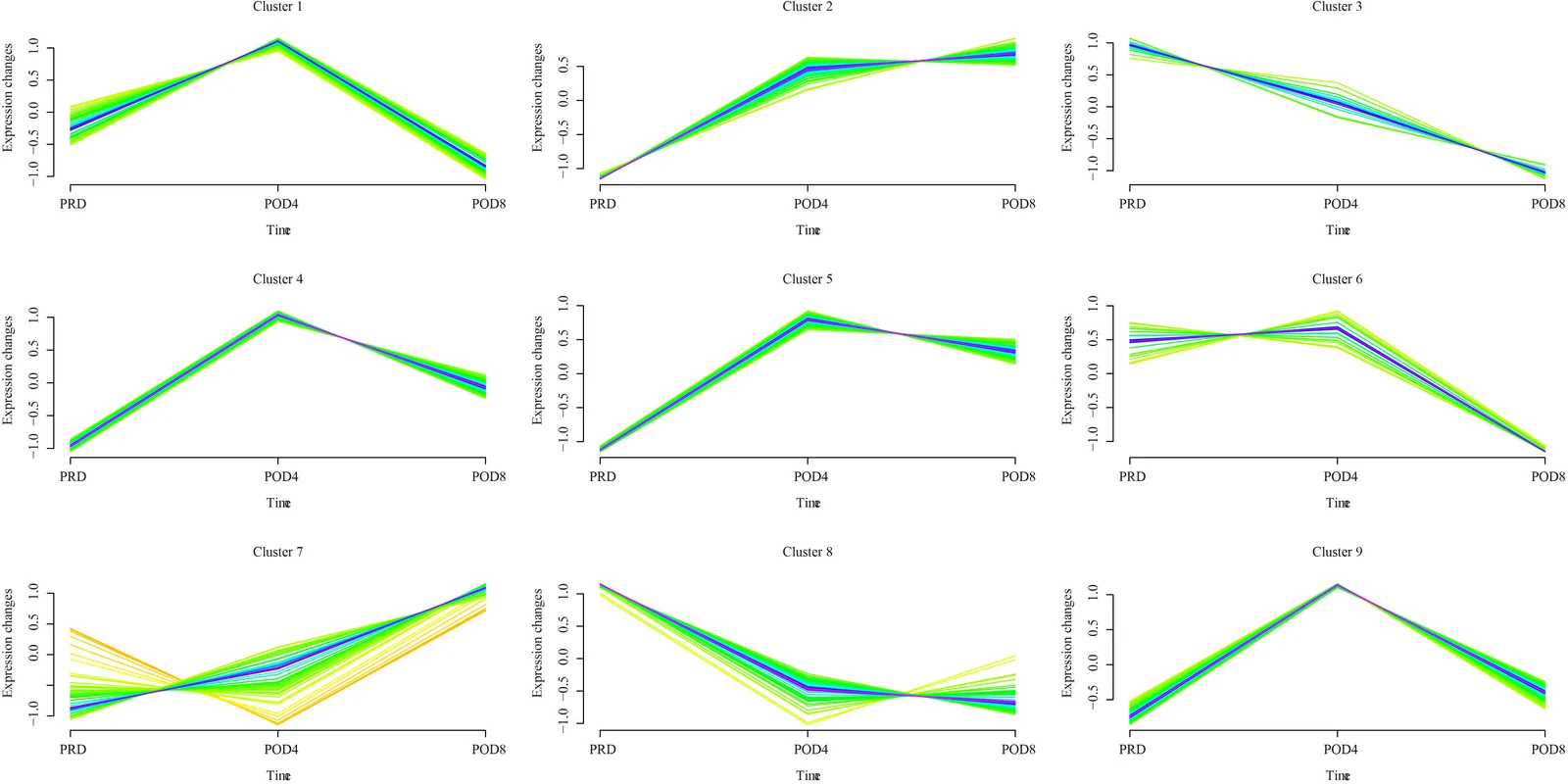
Mfuzz cluster analysis.

Through Mfuzz clustering analysis, we identified nine gene clusters exhibiting distinct expression dynamics, as shown in **Figure 2**. These clusters clearly reveal the temporal expression patterns of genes in colorectal cancer (CRC) patients across three time points: preoperative (PRD), early postoperative (POD4), and recovery phase (POD8). Specifically, genes in Clusters 1, 4, 5, 6, and 9 showed significant upregulation at POD4, followed by a decline at POD8. In contrast, gene expression in Clusters 3 and 8 exhibited sustained downregulation postoperatively, remaining at low levels by POD8. These findings reveal temporal changes in protein module expression accompanying CRC progression, suggesting these proteins hold significant potential as prognostic biomarkers for CRC.

### Functional and pathway enrichment analysis of differentially expressed proteins

**Figure 3:**
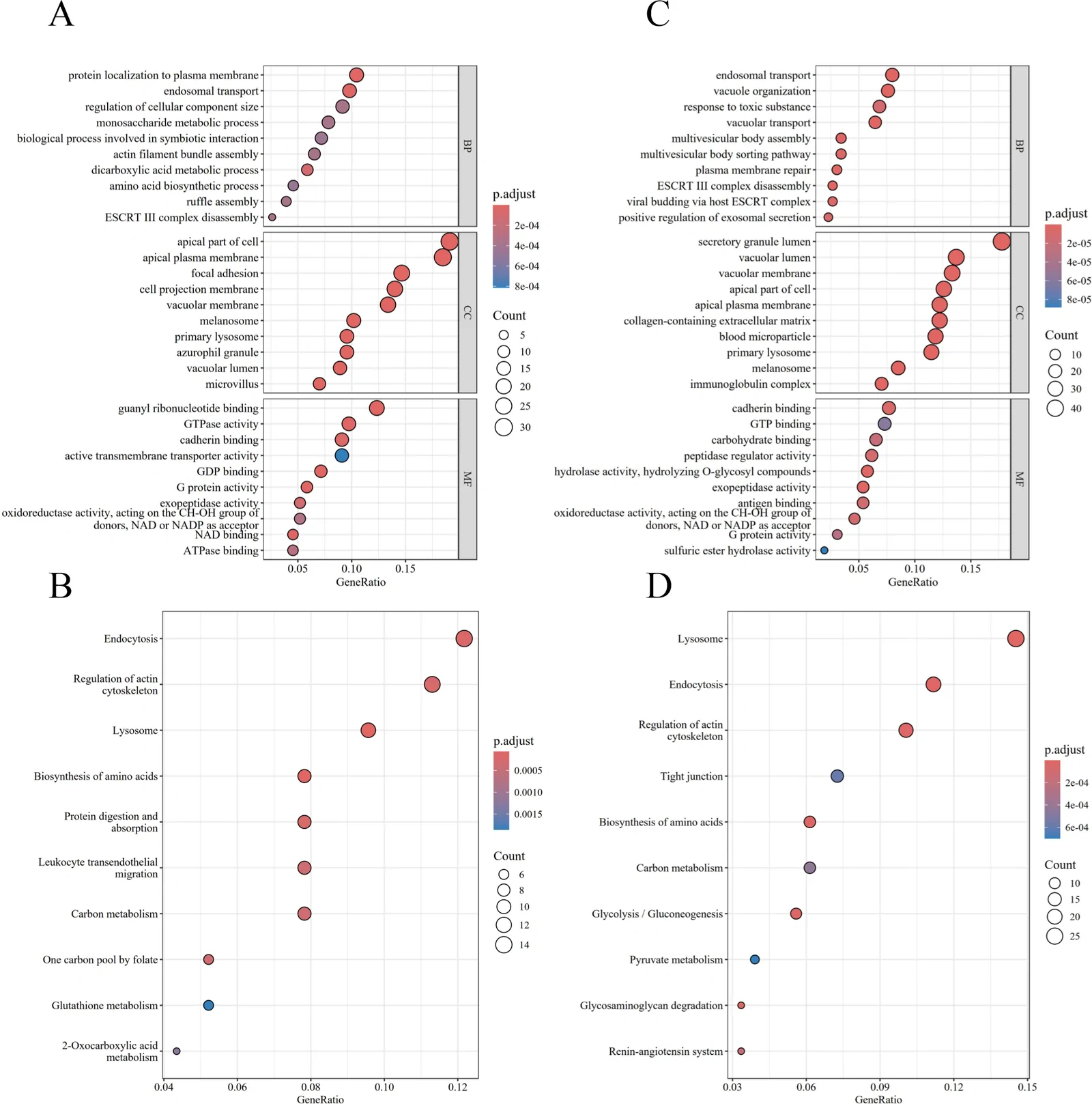
GO and KEGG analysis of differentially expressed proteins. **(A)** GO enrichment analysis of proteins differing between PRD and POD4. **(B)** KEGG Pathway Enrichment Analysis of Differentially Expressed Proteins in PRD vs POD4. **(C)** GO enrichment analysis of proteins differing between PRD and POD8. **(D)** KEGG Pathway Enrichment Analysis of Differentially Expressed Proteins in PRD vs POD8.

Differential proteins between PRD and POD4 are primarily enriched in biological processes involving protein localization to the plasma membrane, endosomal transport, and amino acid metabolism. Their molecular functions include G protein activation and cadherin binding (**Figure 3A**). KEGG pathway analysis reveals significant enrichment in pathways such as amino acid biosynthesis, lysosomal processes, endocytosis, and actin cytoskeleton regulation (**Figure 3B**).

Differential proteins between PRD and POD8 showed significant enrichment in biological processes related to endosomal transport, vacuolar organization, and stress responses, while molecular functions were enriched in cadherin and GTP binding (**Figure 3C**). KEGG pathway analysis indicates significant enrichment in pathways including lysosomal processes, endocytosis, actin cytoskeleton regulation, tight junctions, amino acid and carbohydrate metabolism, and the renin-angiotensin system (**Figure 3D**).

### Analysis of stable downregulated proteins following colorectal cancer surgery

**Figure 4:**
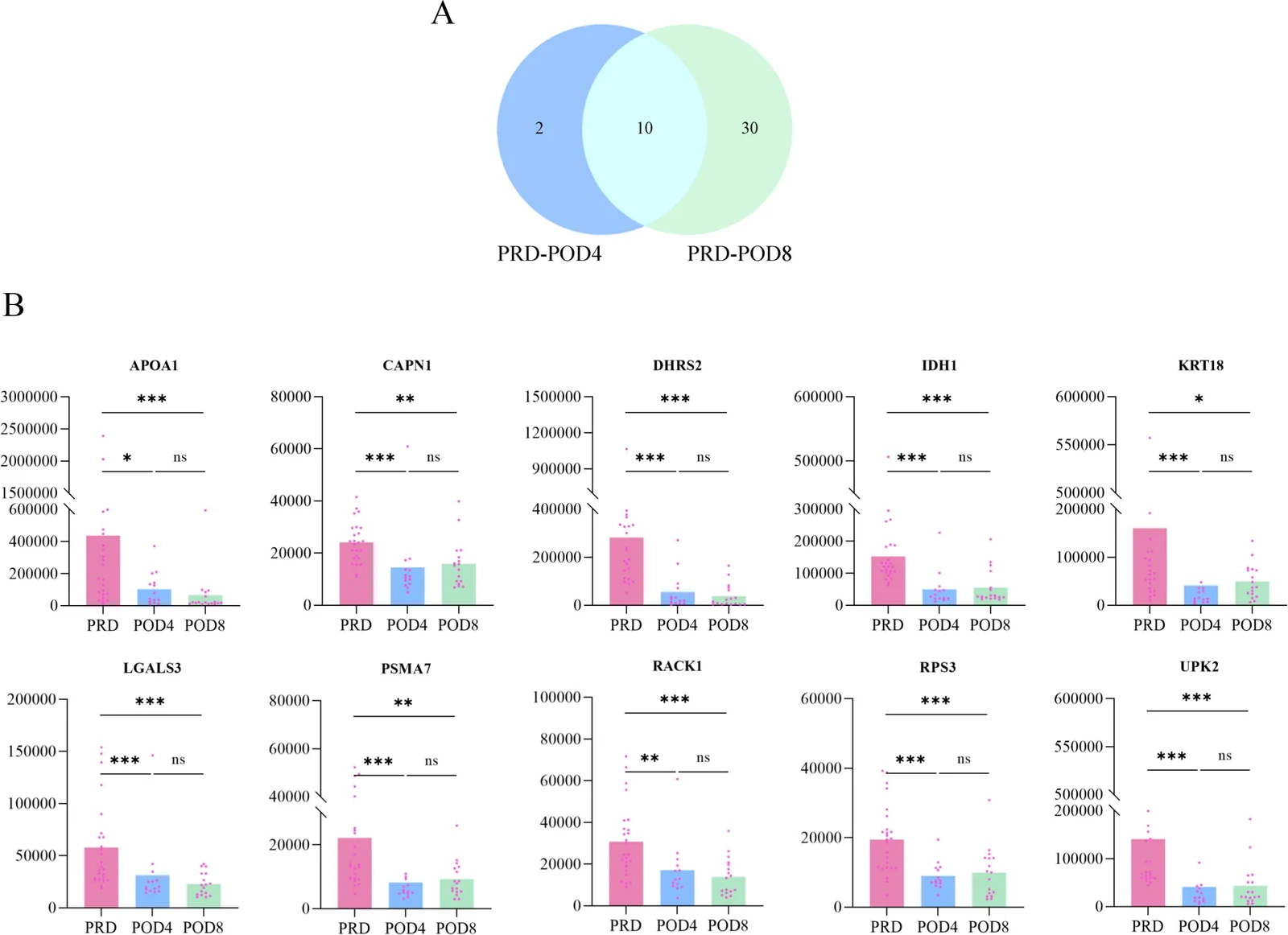
Analysis of postoperative downregulation of protein expression. **(A)** Venn diagram. **(B)** Postoperative CRC downregulates ten stable protein expression levels. ns, P > 0.05; *, P < 0.05; **, P < 0.01; ***, P < 0.001.

To identify stably downregulated proteins, we compared the differentially expressed protein profiles between the PRD group and both the POD4 and POD8 groups. Through Venn diagram analysis, we identified 10 proteins that were significantly downregulated in both comparisons (*P* < 0.05, **Figure 4A**). These included keratin 18 (KRT18), proteasome subunit alpha 7 (PSMA7), isocitrate dehydrogenase 1 (IDH1), 40S ribosomal protein S3 (RPS3), calcium-activated proteinase 1 (CAPN1), urokinase-like protease 2 (UPK2), receptor activator of nuclear factor kappa-B 1 (RACK1), galectin-3 (LGALS3), short-chain dehydrogenase/reductase 2 (DHRS2), and apolipoprotein A1 (APOA1). Subsequently, we plotted mass spectrometry data statistics for these 10 key proteins (**Figure 4B**) to visually demonstrate their expression levels across groups.

### Protein correlation analysis with CRC surgery with stable postoperative results

**Figure 5:**
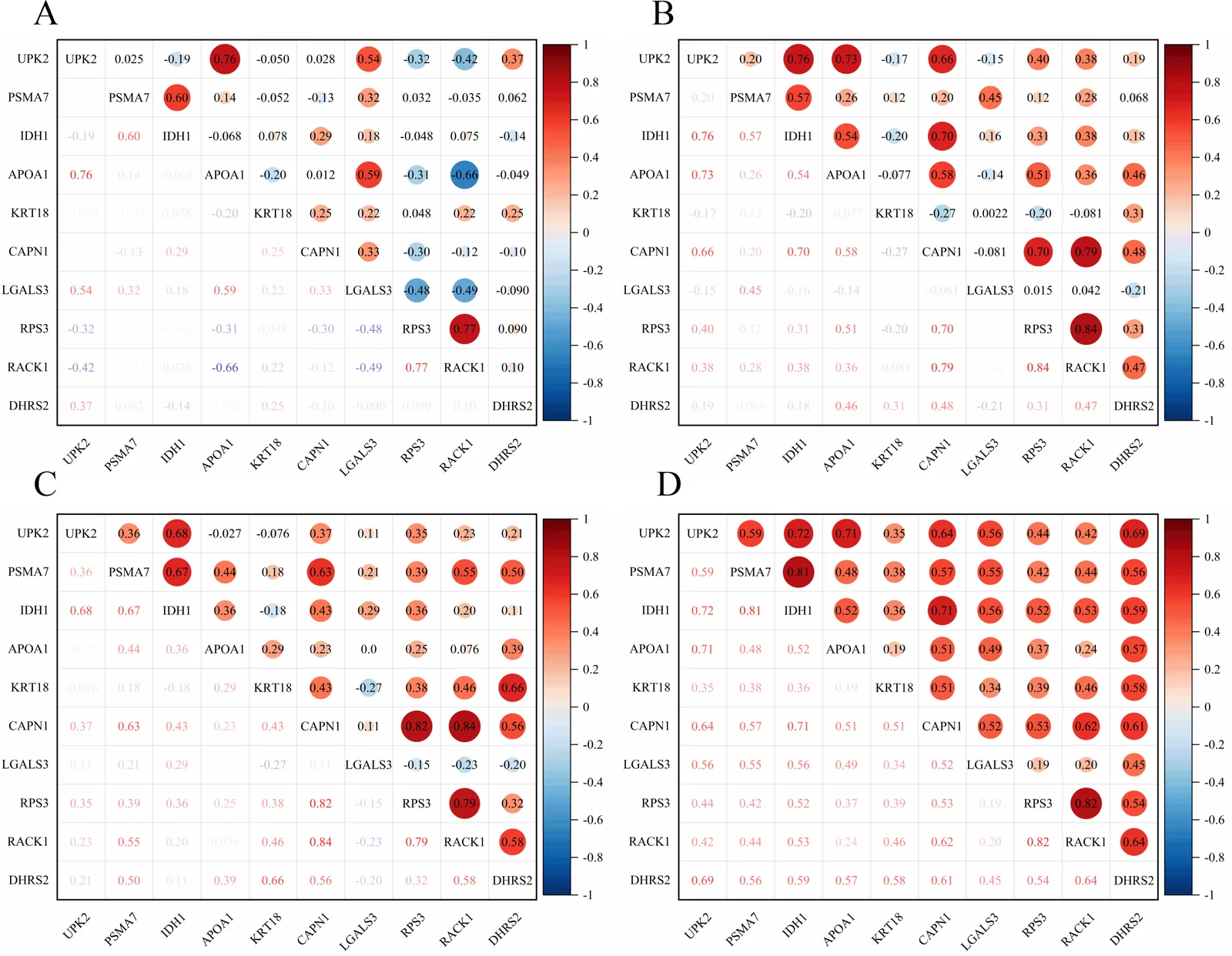
Postoperative stable downregulation of protein expression correlation heatmap. **(A)** PRD Group. **(B)** POD4 Group. **(C)** POD8 Group. **(D)** PRD, POD4, and POD8 groups.

To investigate the interactions among ten downregulated stabilizing proteins, we analyzed their correlations within the PRD, POD4, and POD8 subgroups as well as across the entire dataset. Results revealed that only two protein pairs showed consistent correlations throughout all analyses: PSMA7 and IDH1, and RPS3 and RACK1. Within the PRD group, PSMA7 correlated with IDH1 (*r* = 0.598, *p* = 0.002), and RPS3 correlated with RACK1 (*r* = 0.766, *p* < 0.001, **Figure 5A**). In the POD4 group, PSMA7 correlated with IDH1 (*r* = 0.569, *p* = 0.034), and RPS3 correlated with RACK1 (*r* = 0.837, *p* < 0.001, **Figure 5B**). In the POD8 group, PSMA7 correlated with IDH1 (*r* = 0.674, *p* = 0.003), and RPS3 correlated with RACK1 (*r* = 0.787, *p* < 0.001, **Figure 5C**). A comprehensive analysis of data across all three groups further confirmed (**Figure 5D**) PSMA7 and IDH1 (*r* = 0.814, *p* < 0.001) as well as RPS3 and RACK1 (*r* = 0.820, *p* < 0.001) as core protein pairs exhibited sustained and highly consistent synergy throughout the process, suggesting they may function as units jointly participating in disease-related regulatory pathways.

### Postoperative assessment of diagnostic performance of stable downregulation of IDH1, PSMA7, RPS3, and RACK1 in CRC

**Figure 6:**
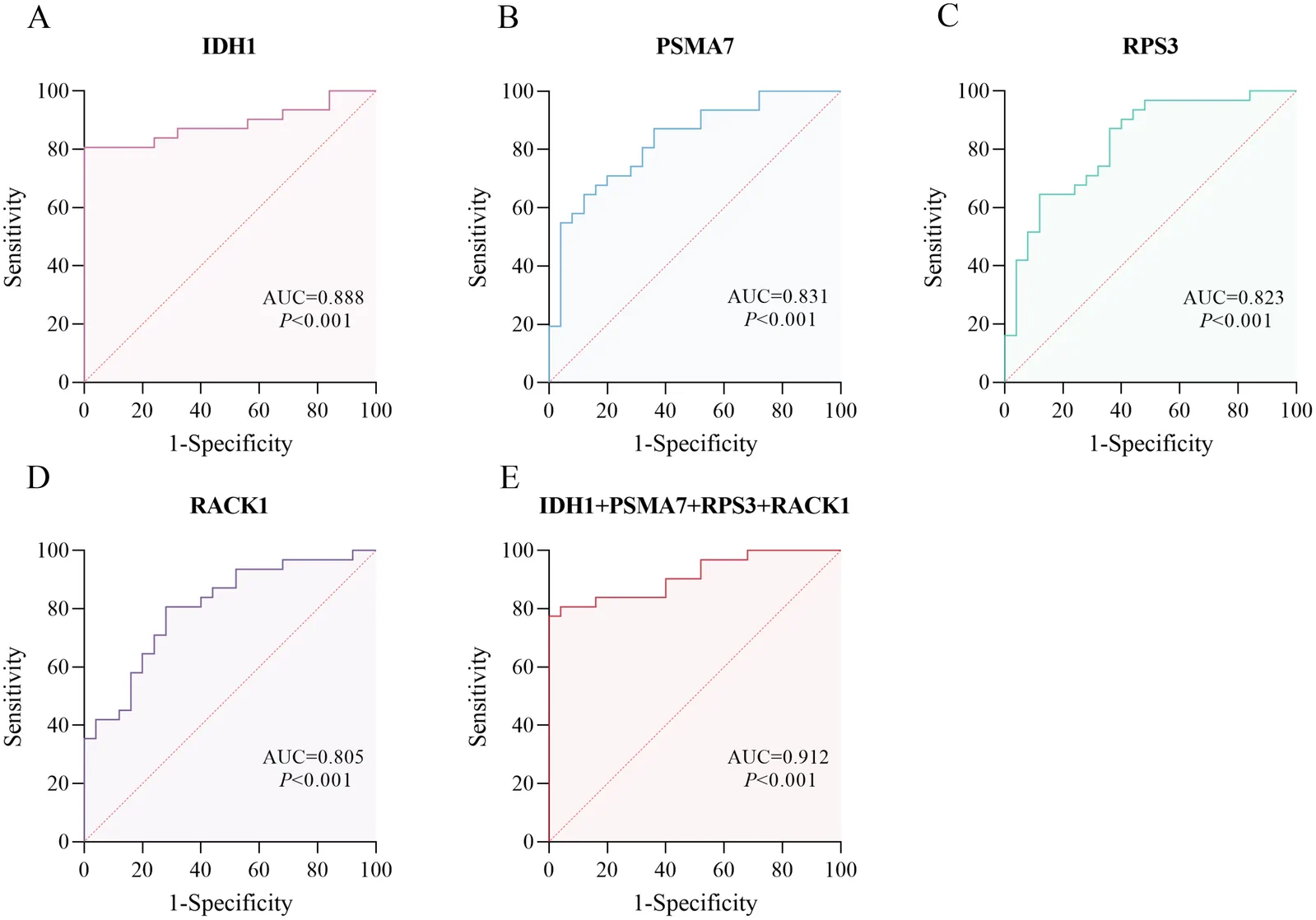
Assessment of diagnostic performance. **(A)** ROC curve for IDH1. **(B)** ROC curve for PSMA7. **(C)** ROC curve for RPS3. **(D)** ROC curve for RACK1. **(E)** ROC curve for the combined use of IDH1, PSMA7, RPS3, and RACK1.

To evaluate the diagnostic value of RACK1, PSMA7, RPS3, and IDH1 for CRC prognosis, we combined the POD4 and POD8 groups into a postoperative cohort and performed ROC curve analysis against the preoperative cohort. The combined diagnostic model was constructed using binary logistic regression, with the expression levels of the four proteins serving as independent variables. The predicted values of each indicator were integrated through weighted combination to generate a composite predicted probability, which was subsequently used for ROC curve analysis. Results demonstrated that all four proteins—PSMA7, IDH1, RACK1, and RPS3—exhibited excellent diagnostic performance. IDH1 (AUC = 0.888, *P* < 0.001, **Figure 6A**), PSMA7 (AUC = 0.831, *P* < 0.001, **Figure 6B**), RPS3 (AUC = 0.823, *P* < 0.001, **Figure 6C**), and RACK1 (AUC = 0.805, *P* < 0.001, **Figure 6D**). Notably, the AUC further increased to 0.912 (*P* < 0.001, **Figure 6E**) when all four markers were used in combination, indicating higher diagnostic accuracy with joint detection.

## Discussion

In the clinical management of colorectal cancer, monitoring prognostic biomarkers is crucial for guiding treatment decisions, assessing disease progression, and predicting survival. Recent advances in molecular diagnostic technologies have driven the discovery of novel biomarkers. For instance, circulating DNA methylation markers such as SEPTIN9, SDC2, and BCAT1 (21, 22) have been demonstrated to significantly correlate with patient survival and dynamically reflect tumor biological behavior. Additionally, radiomics indicators based on CT body composition scores (such as skeletal muscle index, SMI) offer new perspectives for prognostic assessment (23, 24), demonstrating significant associations with overall survival (OS) and disease-free survival (DFS). Existing biomarkers still face challenges in clinical application, including limited specificity, insufficient standardization of testing protocols, and the complexity of combining multiple indicators (25, 26). Currently, there is a clinical shortage of biomarkers capable of accurately and non-invasively monitoring postoperative recovery in patients. Therefore, identifying novel biomarkers with high specificity and sensitivity that can reflect real-time recovery of postoperative physiological homeostasis holds significant practical importance for improving the clinical management of colorectal cancer patients.

This study employed label-free quantitative proteomics to systematically analyze urinary proteomic profiles in colorectal cancer (CRC) patients during preoperative, early postoperative, and recovery phases, aiming to identify stably downregulated proteins closely associated with postoperative recovery. We identified 3,695 proteins across the three sample groups. Differential expression analysis revealed 161 and 277 differentially expressed proteins in the POD4 and POD8 groups, respectively, compared to the preoperative group, indicating that surgery induced significant proteomic reprogramming. More importantly, Mfuzz temporal clustering analysis revealed distinct gene expression dynamics, particularly in Clusters 3 and 8, where proteins exhibited sustained postoperative downregulation, remaining at low levels even at POD8. This phenomenon suggests that the levels of these urinary proteins are closely associated with the removal of tumor burden *in vivo*, demonstrating potential value as biomarkers for monitoring postoperative recovery and assessing prognosis in CRC patients.

**Figure 7:**
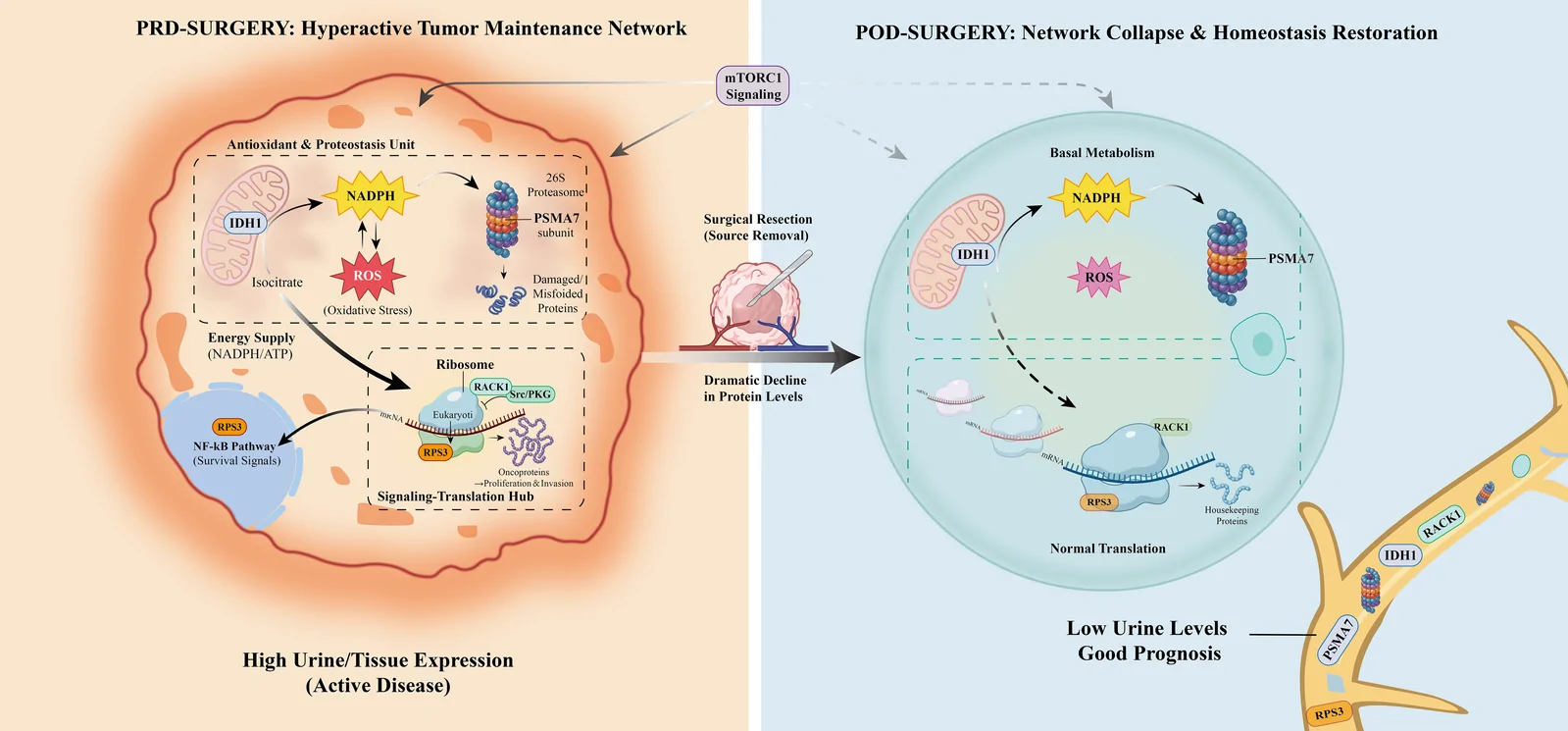
Schematic diagram of the synergistic interaction network of PSMA7, IDH1, RPS3, and RACK1.

This study revealed that PSMA7-IDH1 and RPS3-RACK1 proteins in postoperative urine from male CRC patients exhibited persistent and highly consistent synergistic downregulation. This not only reflects tumor burden clearance but also reveals the systemic collapse of the intricate molecular network upon which cancer cells rely to maintain their malignant phenotype post-surgery. As shown in **Figure 7**, this coordinated alteration of the protein pair may be associated with the mTORC1 signaling pathway (27). On one hand, IDH1 counteracts oxidative stress within cells by generating NADPH (28) and, together with PSMA7, maintains protein homeostasis during rapid tumor cell growth (29); on the other hand, RPS3 and RACK1 form a signaling hub on ribosomes, promoting the synthesis of oncoproteins (30, 31). Crucially, the PSMA7-IDH1 module provides energy and material support for the RPS3-RACK1 module, with both being tightly interconnected. Consequently, the synchronized decline of these two protein pairs in urine following surgical tumor resection indicates disruption of the malignant maintenance network in cancer cells and the body’s return to normal physiological states. This finding suggests that the aforementioned proteins may serve as candidate biomarkers to assist in monitoring the physiological recovery process following surgery in male patients with CRC. Nevertheless, their association with postoperative residual disease status and prognosis warrants further validation through integration with imaging assessment, pathological margin status, and long-term follow-up data.

This study has several limitations. First, only male patients with colorectal cancer were enrolled, and the overall sample size was relatively limited, precluding an investigation of the influence of sex differences on the dynamic changes in perioperative urinary proteomic profiles. Furthermore, although the study groups demonstrated good comparability with respect to key clinical characteristics, including tumor location, pathological staging, and degree of differentiation, the current sample size was insufficient to support more in-depth subgroup analyses stratified by these clinicopathological features. Future studies should seek validation in larger, multicenter, independent cohorts encompassing patients of both sexes, particularly females, and should incorporate more comprehensive clinical classifications to facilitate further investigation into the effects of tumor location, stage, and grade, thereby improving the generalizability of the findings. Second, the specific molecular interactions underlying the PSMA7–IDH1 and RPS3–RACK1 axes, as well as their precise mechanistic roles in tumor recurrence, require further confirmation through both *in vitro* and *in vivo* experimental approaches. The combined ROC model presented in this study was constructed based on the current sample and has not yet undergone internal cross-validation or external independent cohort validation; therefore, its generalization capacity warrants further evaluation. Additionally, the present study compared urinary proteomic differences across surgical stages solely at the population level, and future investigations should further validate the clinical applicability of the identified candidate biomarkers in paired cohorts or larger independent validation cohorts. In conclusion, this study characterized the dynamic alterations in urinary protein expression before and after surgery in patients with colorectal cancer, and identified a four-protein combined biomarker panel consisting of IDH1, PSMA7, RPS3, and RACK1 with high diagnostic performance. These findings provide a novel strategy for the development of noninvasive and dynamic postoperative monitoring tools for CRC.

## Conclusion

Through label-free quantitative proteomics combined with bioinformatic analysis, this study systematically characterized the differential changes in urinary proteomic profiles across the preoperative and distinct postoperative stages in male patients with colorectal cancer. The results demonstrated that IDH1, PSMA7, RPS3, and RACK1 exhibited a sustained downregulation trend at multiple time points following tumor resection. These proteins may serve as a panel of candidate noninvasive biomarkers to assist in the monitoring of postoperative recovery and clinical assessment, although their clinical utility warrants further validation in large-scale independent cohorts.

## Data Availability

The original contributions presented in the study are included in the article. Further inquiries can be directed to the corresponding author.
